# An Incarcerated Inguinal Hernia Masking a Reactive Nodular Fibrous Pseudotumor: A Case Report

**DOI:** 10.7759/cureus.73027

**Published:** 2024-11-04

**Authors:** Ismini Kountouri, Ioannis Katsarelas, Dimitrios Chatzinas, Miltiadis Chandolias, Periklis Dimasis

**Affiliations:** 1 Department of General Surgery, General Hospital of Katerini, Katerini, GRC; 2 Department of Surgery, General Hospital of Katerini, Katerini, GRC

**Keywords:** emergency surgery, hartmann’s operation, incarcerated inguinal hernia, rare tumors, reactive nodular fibrous pseudotumor

## Abstract

A reactive nodular fibrous pseudotumor (RNFP) is a rare benign tumor that is related to previous abdominal surgery, injury, or inflammation, and in patients with a history of malignancy; it is easily misdiagnosed as tumor recurrence or metastasis. We discuss the case of an 80-year-old patient who presented to the emergency department with a large non-reducible inguinal hernia. The patient was taken to the operating room for an open hernia repair through an inguinal incision. Perioperative findings were a large left-sided inguinal hernia, which contained the sigmoid colon with a gross solid mass emerging from the bowel wall. The mass was infiltrating the left testicle and resection of the sigmoid colon, the mesentery, and the left testicle, without a primary anastomosis, was performed through the inguinal incision. A sigmoid stoma was installed and the abdominal wall was restored according to the Lichtenstein technique. The histopathology results revealed an RNFP arising from the sigmoid colon. To our knowledge, this is the first report of such a tumor created on the ground of chronic fibrosis from an inguinal hernia. Discovery of an RNFP during laparotomy for malignant tumors is generally reported in the literature, especially for patients with a history of cancer. Our report showcases that surgeons should remain vigilant regarding these tumors in patients with chronic inguinal hernias and masses found inside the hernia sacs.

## Introduction

A reactive nodular fibrous pseudotumor (RNFP) is a reactive benign lesion that results from injury or inflammation and appears as a tumor-like lesion characterized by reactive fibroblast and myofibroblast proliferation within collagenic hyalinized stroma [1]. These tumors are usually associated with the gastrointestinal (GI) tract and peritoneal regions. They can present as a single mass or multiple nodules attached to the outer layer of the bowel wall, mesentery, or omentum [1,2]. RNFPs generally have a good prognosis with no cases of recurrence or metastasis reported in the literature [1]. They belong to the heterogeneous group of fibroinflammatory tumors, or inflammatory pseudotumors that includes distinct entities like calcifying fibrous pseudotumors, gastrointestinal stromal tumors (GIST), inflammatory myofibroblastic (IMF) tumors, inflammatory fibrosarcoma, sclerosing mesenteritis, and other non-neoplastic fibrous proliferations [3]. To our knowledge, this is the first report of such a tumor created on the ground of chronic fibrosis from an inguinal hernia.

## Case presentation

**Figure 1:**
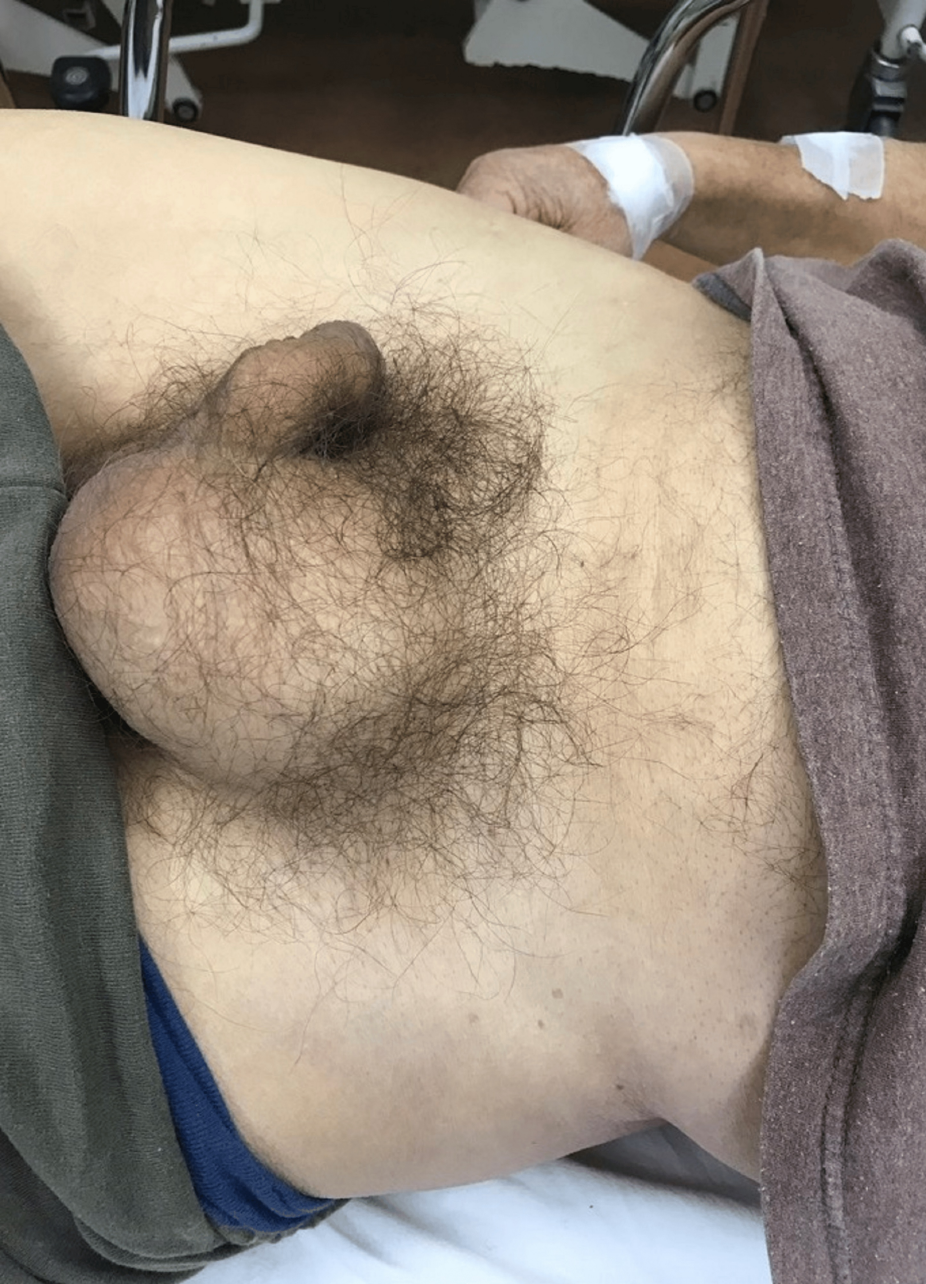
Non-reducible left inguinal hernia in the patient.

We discuss the case of an 80-year-old man who presented to the Emergency Department of the General Hospital of Katerini, in Greece, complaining of acute pain in his left inguinal area that started two hours ago. Upon clinical examination, the patient was hemodynamically stable and a large non-reducible left inguinal hernia was discovered (Figure 1). The patient admitted to having a left inguinal hernia for 20 years and not being able to reduce it for almost 24 hours before coming to the hospital. He complained of no constipation or presence of blood in his stool and his abdomen showed no tenderness upon palpation. He was then admitted to the Surgical Department and taken to the operating room for an open hernia repair through an inguinal incision.

**Figure 2:**
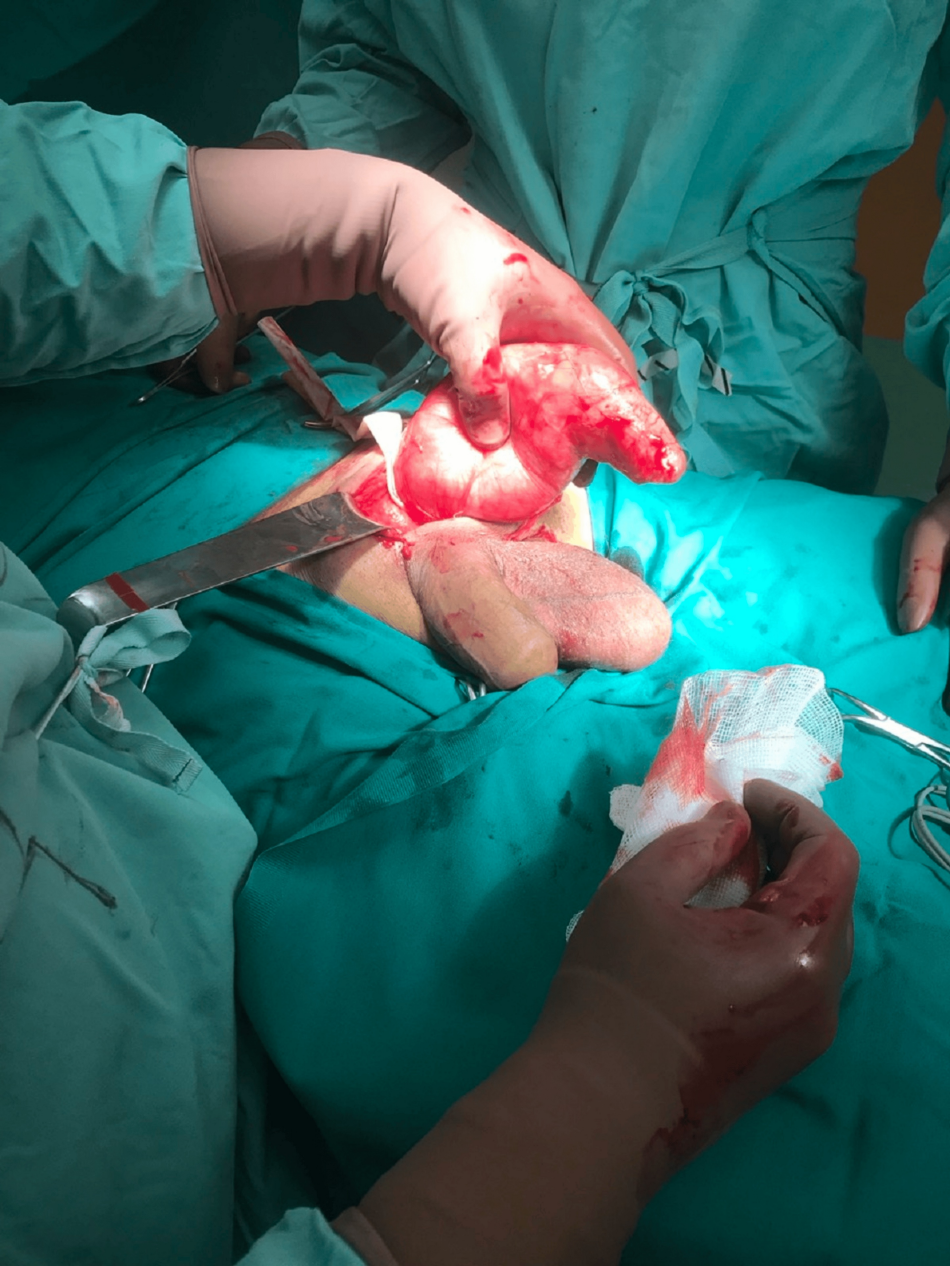
Perioperative image showing the sigmoid colon with a large solid mass arising from the bowel wall. The left testicle has already been resected to facilitate the mobilization of the colon with the tumor.

Perioperative findings were a large left-sided inguinal hernia that contained the sigmoid colon with a gross solid mass emerging from the bowel wall (Figure 2). The mass was infiltrating the left testicle, prompting a resection of the sigmoid colon, mesentery, and left testicle without performing a primary anastomosis. This procedure was conducted through an inguinal incision.

**Figure 3:**
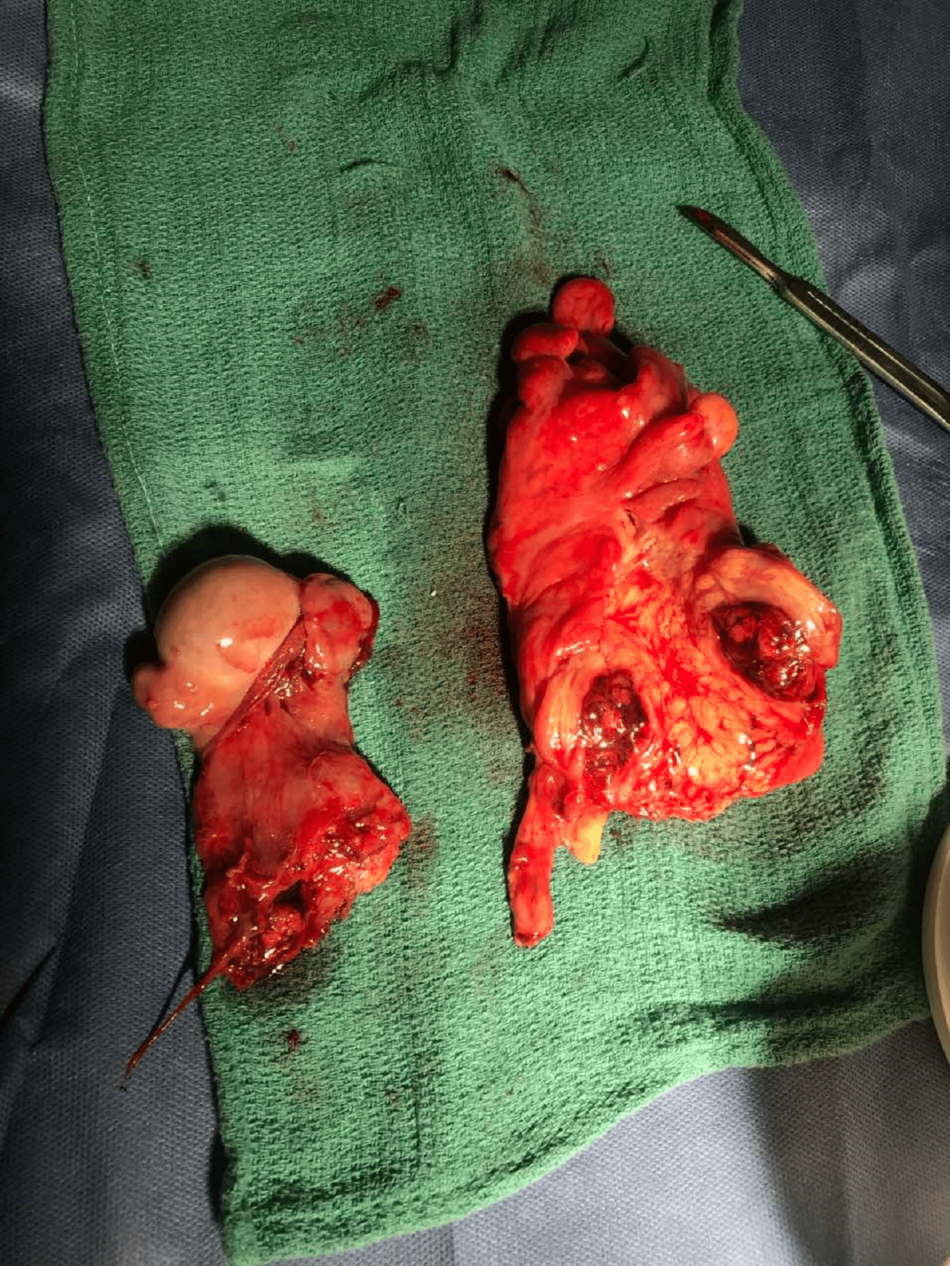
The specimen of the left testicle and the sigmoid colon with the tumor protruding for the bowel wall that was sent for histopathological examination.

The specimen of the bowel and testicle excision was sent for histopathological examination (Figure 3). A sigmoid stoma was installed and the abdominal wall was restored according to the Lichtenstein technique. The patient was discharged in good clinical condition four days after surgery and waited for the histopathology results. The histopathology results revealed an RNFP arising from the sigmoid colon. The proliferative spindle cells were disorderly arranged, with a small amount of interstitial lymphocyte infiltration. Immunohistochemical analysis revealed an expression of vimentin and smooth muscle actin (SMA) in most spindle cells while staining for S100 proteins, CD117 (c-kit), DOG1, P63, CK (Pan), or anaplastic lymphoma kinase (ALK-1 A4) were found negative. To our knowledge, this is the first report of such a tumor created on the ground of chronic fibrosis from an inguinal hernia.

Excision was complete and a bowel anastomosis was performed a year later. No evidence of recurring disease was found during a three-year observation period.

## Discussion

First described by Yantiss et al. in 2003, RNFPs are identified by a rich in collagen, wire-like, keloidal, or hyalinized stroma [2]. Intralesional mononuclear cells are rare but more frequent peripherally and arranged in lymphoid aggregates [2]. These tumors are usually associated with the GI tract and peritoneal regions. They can present as a single mass or multiple nodules attached to the outer layer of the bowel wall, mesentery, or omentum [1,2].

RNFPs generally have a good prognosis with no cases of recurrence or metastasis reported in the literature [1]. They belong to the heterogeneous group of fibroinflammatory tumors, or inflammatory pseudotumors that includes distinct entities like calcifying fibrous pseudotumors, GIST, IMF tumors, inflammatory fibrosarcoma, sclerosing mesenteritis, and other non-neoplastic fibrous proliferations [3]. RNFPs can easily be misdiagnosed as more aggressive types of tumors, such as metastatic malignant neoplasms, primary GISTs, or IMF tumors, and should be considered in the differential diagnosis of primary and metastatic GI tumors [1].

Histopathological examination of RNFP reveals a proliferation of stellate or spindle cells with a keloid-like appearance in a dense collagenous background, accompanied by infiltration of lymphocytes and plasma cells, just like in our case [4-6]. Immunohistochemistry is positive in staining for vimentin and SMA and negative for CD34 and S-100 [4-6]. Imaging scans can be helpful in identifying these tumors. These lesions appear isodense on a plain computed tomography (CT) scan, with progressively mild enhancement observed on postcontrast CT [5]. In magnetic resonance imaging (MRI), the lesion shows homogeneous hypointensity on T1 weighted image (T1WI) and a mixed high-signal shadow with a slight hypointensity area on T2WI, while enhancement mode results are the same as those by CT [5].

Even though these cases of RNFPs are rare they are an important finding as they can lead to a misdiagnosis of a more aggressive type of tumor, such as metastatic malignant neoplasm, primary GIST, or IMF tumor [1]. Ultrastructural evaluation can aid in the differential diagnosis of GISTs. This method typically reveals GIST cells with characteristic long interdigitating processes, intermediate filaments, solitary focal densities, and attachment plaques with incomplete external lamina, which are crucial for accurate diagnosis [7]. Surgical management is generally the treatment of choice for these tumors [5]. No cases of RNFP recurrence or metastasis have been reported to date [5]. As this was a case of an intravascular tumor, which is a primary tumor of abdominal organs within a hernia sac [8], the choice of the abdominal versus inguinal approach depends on the patient’s anatomy, the surgical findings, and the surgeon’s experience [8]. In 2024, Baldi et al. reported 83 cases of sigmoid colon malignancy inside an incarcerated inguinal hernia [8]. In cases where a physical examination reveals a non-reducible inguinal groin mass, surgery may be performed in an emergency environment, and as a result, these carcinomas are often found as incidental findings, resulting in suboptimal treatment [8].

To our knowledge, this is the first report of an RNFP inside an incarcerated inguinal hernia. Other cases of such tumors, mainly arising within the abdominal cavity, including the GI tract, mesentery, and retroperitoneum have been reported in the literature. In the majority of the cases, RNFPs were initially misdiagnosed and the final diagnosis was installed after the histopathological examination.

In 2015, Yan et al. reported on a 60-year-old female patient with RNFP involving the GI tract and mesentery. In this case, the patient presented with abdominal pain that had gradually developed over six months following abdominal surgery for leiomyoma of the uterus. Upon laparoscopy, multiple nodules diffused throughout the abdominal cavity were identified and misdiagnosed as metastatic cancer. Microscopic examination revealed that the lesion was composed of spindle or stellate cells resembling fibroblasts/myofibroblasts enmeshed in a collagenous matrix [1].

In 2022, Chen et al. reported on an RNFP mimicking metastatic tumor after a gastric cancer operation. Abdominal CT showed a solid mass measuring 5.0 cm × 5.2 cm in the operative area of gastric cancer with a relatively clear boundary, isodensity, and no necrosis. After excision, the histopathological examination also revealed the presence of an RNFP [5].

In 2005, Saglam et al. reported on a 28-year-old woman with a history of dysmenorrhea and ergotamine use for migraine attacks who presented with abdominal pain and bilateral cystic lesions in the ovaries, which were thought to be consistent with endometrioma on pelvic ultrasonography. After excision, the histopathology results revealed numerous fibrocollagenous nodules, involving the peritoneum of the abdominal and pelvic cavities, possibly secondary to endometriosis and/or ergotamine use [9].

In 2012, McAteer et al. described a case of a 13-year-old girl presenting with acute abdominal pain and imaging suggesting acute appendicitis with a mass attached to the jejunum that had torsed upon its blood supply. Histopathology revealed that the mass to be an RNFP [10].

In 2018, Moodley et al. reported on an RNFP mimicking a metastatic GIST to the perigastric lymph nodes. Immunohistochemical workup supported the myofibroblastic origin of the spindle cells, consistent with an RNFP, and definitively excluded metastatic GIST [11].

In 2014, Yi et al. reported on an RNFP in the lesser curvature of the fundus attached to the left adrenal gland with obscure boundaries. Immunohistochemically, the biopsy specimen was positive for CK and M-CEA in the glandular epithelium, positive for CD31 and CD34 in the vascular endothelium, and positive for CD3 and L26 in small lymphocytes; therefore, lymphoma and gastric cancer were excluded [12].

Since these tumors belong to the heterogenous group of fibroinflammatory tumors, we can safely conclude that in our case the tumor was a result of the inflammatory infiltration, vascular damage, and regressive nerve lesions that usually are present in the tissues inside an inguinal hernia [13]. Thus, all patients with incarcerated inguinal hernias can eventually develop such tumors inside the hernia sacks.

## Conclusions

Even though our case report regards a benign tumor, it showcases that sometimes an incarcerated inguinal hernia may mask a colonic tumor. In our case, a simple excision of the tumor was sufficient. Still, in cases of sigmoid colon carcinomas a more excessive colectomy, according to the oncological criteria, should be performed. With our case report, we intend to raise vigilance in surgeons regarding cases of incarcerated inguinal hernias that may contain sigmoid colon tumors, either benign or malignant. Surgeons should keep in mind that when operating on non-reducible inguinal hernias masking colonic tumors, a risk of ineffective care should be avoided for the optimal result for the patient.
